# Outcomes of Bolus Dose Furosemide Versus Continuous Infusion in Patients With Acute Decompensated Left Ventricular Failure and Atrial Fibrillation

**DOI:** 10.1002/clc.70033

**Published:** 2024-11-18

**Authors:** Babar Khan, Darab Shuja, Burhan ul Haq Saqib, Maria Liaquat, Jahanzeb Malik, Amna Ashraf, Ali Karim, Iftikhar Ahmed, Waheed Akhtar, Amin Mehmoodi

**Affiliations:** ^1^ Department of Cardiology Lady Reading Hospital Peshawar Pakistan; ^2^ Department of Medicine Services Hospital Lahore Pakistan; ^3^ Department of Cardiology DHQ Hospital Mirpur Pakistan; ^4^ Department of Medicine Benazir Bhutto Hospital Rawalpindi Pakistan; ^5^ Department of Cardiovascular Medicine Cardiovascular Analytics Group Islamabad Pakistan; ^6^ Department of Medicine Military Hospital Rawalpindi Pakistan; ^7^ Department of Medicine Liaquat University of Medical and Health Sciences Jamshoro Pakistan; ^8^ Department of Cardiology Abbas Institute of Medical Sciences Muzaffrabad Pakistan; ^9^ Department of Medicine Ibn e Seena Hospital Kabul Afghanistan

**Keywords:** acute decompensated heart failure, diuretic regimens, furosemide, hypertonic saline solution, renal function

## Abstract

**Background:**

This prospective, randomized trial aimed to compare the efficacy and safety of different intravenous diuretic regimens in acute decompensated heart failure (ADHF) patients.

**Methods:**

ADHF patients were enrolled and randomized into three groups: continuous intravenous furosemide infusion (cIV), bolus furosemide injection (bI), and furosemide plus hypertonic saline solution (HSS). Clinical outcomes were assessed over 48 h.

**Results:**

In a study involving 1276 patients admitted for ADHF, three therapeutic regimens (T × 1, T × 2, and T × 3) were compared. T × 1 administered an 80 mg furosemide intravenous bolus infusion twice daily to 479 patients, while T × 2 involved a continuous 16‐h infusion of 160 mg furosemide daily to 420 patients. T × 3 treated 377 patients with 160 mg furosemide combined with 150 mL of HSS containing 1.95% NaCl over 30 min. Yet, overall changes in renal markers such as BUN, Na, K, and serum creatinine did not differ significantly. Analysis of prespecified study endpoints revealed notable variations in hospitalization length among the treatment arms. T × 1 demonstrated a significantly shorter hospital stay (3.7 days) compared to T × 2 (6.6 days) and T × 3 (7.9 days). Conversely, alterations in serum creatinine at 48 h, overall changes in serum creatinine, body weight loss, and serum potassium levels did not significantly differ among the treatment groups.

**Conclusion:**

While intravenous bolus of furosemide showed potential benefits in reducing hospitalization duration, limitations such as a small sample size and short‐term observation emphasize the need for larger studies to validate these outcomes further.

## Graphical abstract

While no significant differences were observed in renal function or fluid removal among continuous intravenous furosemide infusion, bolus furosemide injection, and furosemide plus HSS groups, the continuous infusion without HSS exhibited a notably shorter hospital stay. These findings suggest a potential benefit of continuous intravenous furosemide infusion as an effective strategy in reducing hospitalization duration.

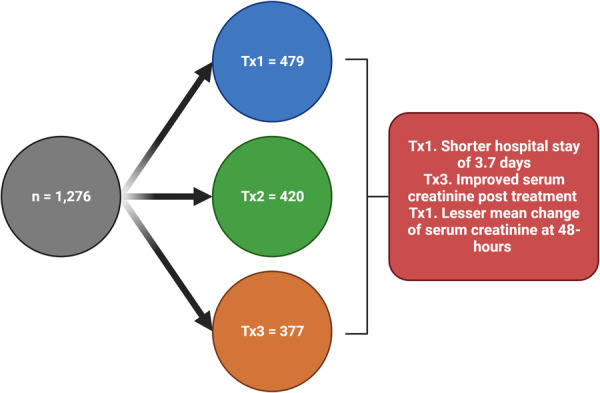

## 1 Introduction

Intravenous loop diuretics serve as a crucial therapeutic approach in managing acute decompensated heart failure (ADHF) with the concurrent presence of atrial fibrillation (AF), aiming to alleviate congestion, enhance oxygenation, and alleviate symptoms [1]. Clinical guidelines highly recommend initiating intravenous diuretic therapy promptly in individuals experiencing ADHF alongside AF, as it improves prognosis and reduces the likelihood of rehospitalization [1, 2]. While rapid decongestion has shown associations with decreased in‐hospital cardiac mortality, it also poses the risk of compromising renal function, leading to prolonged hospital stays and a poorer prognosis, particularly in individuals with the dual challenge of ADHF and AF [3]. Despite recognizing the benefits and potential complications, there is still uncertainty regarding the standardized protocol for administering loop diuretics in ADHF patients with concurrent AF. Dosing and the most effective mode of administration remain unclear. Prior studies have suggested that continuous infusion (cIV) of high‐dose loop diuretics might offer more advantages compared to high‐dose oral or intravenous bolus therapy [4, 5]. However, studies directly comparing bolus injection (bI) with both cIV and furosemide plus hypertonic saline solution (HSS) in the same trial among ADHF patients with AF are lacking. Hence, our goal was to assess and compare the efficacy and safety of these three diuretic regimens specifically in individuals facing the combined challenges of ADHF and AF. The aim was to shed light on the most suitable approach for managing these complex cases and optimizing effectiveness and safety in this patient population.

## 2 Methods

The current study, conducted at a single center (Abbas Institute of Medical Sciences; study ID # AIMS/23/68), was prospective and randomized. It enrolled 1276 patients admitted for ADHF with reduced left ventricular ejection fraction (LVEF), between January 2020 and November 2023, following approval from the institutional ethics committee. All participants provided written informed consent.

Patients admitted to the emergency department within the previous 24 h with ADHF and a pro‐B‐type natriuretic peptide (pro‐BNP) level exceeding 300 pg/mL were included. Exclusion criteria involved individuals who received intravenous diuretics before hospitalization, had serum creatinine levels above 2.0 mg/dL, or systolic blood pressure below 90 mmHg. Additionally, patients needing intravenous vasodilators or inotropic agents besides digoxin, or those suspected of acute coronary syndromes, were excluded.

The therapeutic regimens, labeled T × 1 (bolus infusion), T × 2 (continuous intravenous infusion), and T × 3 (furosemide plus HSS), were prepared before patient enrollment by an independent healthcare team. T × 1 involved giving an 80 mg furosemide intravenous bolus infusion twice daily to 479 patients. T × 2 administered a continuous furosemide infusion of 160 mg for 16 h daily to 420 patients. T × 3 included 160 mg furosemide combined with 150 ml of HSS with 1.95% NaCl and was given over 30 min to 377 patients. All treating physicians were unaware of the administered diuretic regimens. Patients were randomly assigned to the treatment groups through a computer algorithm, independent of the treating physicians, with the HSS concentration chosen based on previous studies to standardize treatment among patients. The study protocol spanned 48 h, after which adjustments to the diuretic regimen were left to the discretion of the treating physicians based on the patient's clinical responses.

Patient demographics, clinical characteristics, laboratory tests including complete blood count, renal function tests, pro‐BNP levels, and echocardiographic measurements were recorded. Endpoints included changes in body weight, serum creatinine levels, and length of hospital stay. Renal dysfunction was defined as a ≥ 0.3 mg/dL increase in serum creatinine from baseline during intravenous diuretic therapy. Laboratory tests involved blood sampling in the morning after fasting, determining blood cell types using an automated count device, and measuring pro‐BNP levels with immunoassay methods. Echocardiography was performed using a specific ultrasound system, calculating ejection fraction using the modified Simpson method.

Statistical analyses were conducted using SPSS software, employing various tests like ANOVA, Kruskal–Wallis, and *χ* ^2^ to compare groups, with significance set at *p* < 0.05. The study adhered to a specific protocol and aimed to evaluate the efficacy and safety of distinct diuretic regimens in ADHF patients, emphasizing changes in weight, renal function, and length of hospital stay as primary outcomes. All statistical analysis was performed on the Statistical Package for Social Sciences (SPSS) version 26 (IBM Corp., Armonk, NY, USA)

## 3 Results

The study enrolled a total of 1276 patients admitted for ADHF and categorized them into three therapeutic regimens: T × 1, T × 2, and T × 3. T × 1 comprised 479 patients who received an 80 mg furosemide intravenous bolus infusion twice daily. T × 2 involved 420 patients receiving a continuous 16‐h infusion of 160 mg furosemide daily. T × 3 included 377 patients who received 160 mg furosemide combined with 150 mL of HSS containing 1.95% NaCl over 30 min. The baseline characteristics showed no statistically significant differences among the treatment groups in various demographic, clinical, and laboratory parameters (Table 1). These included age, gender distribution, body weight, prevalence of diabetes mellitus, hypertension, ischemic etiology, AF, LVEF, baseline BUN, serum creatinine, Na, K, NT‐proBNP levels, hemoglobin, and medication usage (ACE inhibitor or ARB, aldosterone antagonist, β‐blocker, and oral furosemide).

**Table 1:** Baseline characteristics.

Variables	cIV (*n* = 15)	bI (*n* = 14)	HSS (*n* = 14)	*p*
Demographic and clinical data				
Age, years	71.7 ± 10.7	70.6 ± 8.2	65.4 ± 12.2	0.24
Male gender, *n* (%)	7 (50.0)	9 (64.3)	8 (53.3)	0.72
Body weight (kg)	79.0 ± 17.2	85.4 ± 17.3	78.5 ± 18.1	0.57
Diabetes mellitus, *n* (%)	7 (50)	5 (33.3)	9 (64.3)	0.24
Hypertension, *n* (%)	12 (85.7)	11 (73.3)	11 (78.6)	0.71
Ischemic etiology, *n* (%)	4 (28.6)	10 (71.4)	7 (46)	0.24
Atrial fibrillation, *n* (%)	7 (50)	4 (28.6)	5 (33.3)	0.29
LVEF (%)	44.8 ± 9.9	40.5 ± 16.9	41.1 ± 15.7	0.72
Laboratory data				
Baseline BUN (mg/dL)	22.1 ± 7.2	24.1 ± 8.3	25.2 ± 16.5	0.76
Baseline serum creatinine (mg/dL)	0.96 ± 0.29	1.10 ± 0.26	0.93 ± 0.32	0.27
Serum Na (mEq/L)	137.8 ± 4.3	138.3 ± 5.0	136.2 ± 5.2	0.49
NT‐proBNP (pg/mL)	3979 ± 2576	4765 ± 2844	3973 ± 3080	0.51
Hemoglobin (g/dL)	12.4 ± 1.3	12.1 ± 1.5	12.3 ± 2.5	0.88
Medications				
ACE inhibitor or ARB, *n* (%)	9 (64.3)	10 (71.4)	7 (46.7)	0.37
Aldosterone antagonist, *n* (%)	4 (28.6)	2 (14.3)	5 (33.3)	0.47
β‐Blocker, *n* (%)	4 (28.6)	11 (78.6)	9 (60)	0.02
Oral furosemide, *n* (%)	10 (71.4)	9 (64.3)	9 (60)	0.80

Regarding renal function after treatment, the study exhibited differing outcomes across the treatment regimens (Table 2). While serum creatinine levels at 48 h showed statistically significant differences between the groups, the overall changes in serum creatinine, along with other renal function markers such as BUN, Na, and K, did not significantly differ among the treatment groups. Assessing the prespecified study endpoints revealed noteworthy variations in the length of hospitalization among the treatment arms (Table 3). Patients receiving T × 1 had a significantly shorter hospital stay, averaging 3.7 days, compared to 6.6 days for T × 2 and 7.9 days for T × 3 (*p* < 0.01). However, alterations in serum creatinine at 48 h, overall changes in serum creatinine, body weight loss, and serum potassium levels did not demonstrate substantial differences among the treatment groups.

**Table 2:** Renal function tests posttreatment.

Renal function test results after treatment	cIV	bI	HSS	*p*
BUN (mg/dL)	37.9 ± 16.8	38.8 ± 15.3	34.1 ± 18.7	0.72
Serum creatinine at 48 h BUN (mg/dL)	1.17 ± 0.32	0.97 ± 0.27	1.27 ± 0.31	0.04
Serum creatinine BUN (mg/dL)	1.46 ± 0.60	1.27 ± 0.49	1.03 ± 0.40	0.09
Serum Na (mEq/L)	136.0 ± 3.8	134.0 ± 7.2	136.7 ± 4.1	0.37
Serum K (mEq/L)	4.1 ± 0.8	3.9 ± 0.7	4.0 ± 0.9	0.62

**Table 3:** Outcomes.

Prespecified study endpoints	cIV	bI	HSS	*p*
Length of hospitalization (days)	3.7 ± 1.3	6.6 ± 3.4	7.9 ± 4.1	< 0.01
Mean change in serum creatinine at 48 h BUN (mg/dL)	0.04 ± 0.15	0.20 ± 0.21	0.16 ± 0.21	0.08
Mean change in serum creatinine BUN (mg/dL)	0.10 ± 0.28	0.30 ± 0.42	0.36 ± 0.42	0.18
Body weight loss (kg)	5.7 ± 3.6	4.1 ± 2.7	4.6 ± 5.2	0.66

## 4 Discussion

In the treatment of ADHF, furosemide plays a vital role, yet its dosing and mode of administration vary significantly in clinical practice. Few prospective clinical trials have thoroughly explored the effectiveness and safety of diverse furosemide regimens [3, 4, 5]. In our randomized clinical study, we observed no notable differences in renal function, as evaluated by serum creatinine levels, or fluid removal measured by body weight loss, among continuous intravenous furosemide infusion, bolus furosemide injection, and intravenous furosemide with HSS treatment regimens. However, the continuous intravenous furosemide infusion notably correlated with a reduced duration of hospital stay. Ours is among the initial trials comparing all three treatment regimens within a single study. Continuous intravenous infusion of furosemide offers advantages like consistent urine output, leading to a gradual reduction in intravascular volume, reduced neurohormonal activation, less vasoconstriction, and fewer side effects. However, its clinical superiority in terms of efficacy lacks conclusive proof in human studies. Aaser et al. compared cIV with bI in ADHF, finding no difference in urine output and neurohormonal responses [6].

Similarly, in a small study by Copeland et al., no significant differences were observed between groups regarding weight loss, creatinine clearance, serum sodium, potassium changes, or total urine volume over a short duration [7]. Dormans et al. demonstrated that continuous intravenous high‐dose furosemide was more effective than a single bolus in severe heart failure patients [8]. However, a review of multiple studies comparing cIV with bI in heart failure patients showed no significant differences in urine volume or changes in serum creatinine levels, except in a specific study that included HSS. Felker et al. compared diuretic strategies in ADHF patients, where no significant differences in serum creatinine were observed among different dosing methods [9]. Another study by Allen et al. comparing bI with cIV showed no significant differences in patient outcomes between the two methods up to the third day of hospitalization or discharge [10]. Studies evaluating intravenous furosemide plus HSS indicated reductions in hospital stay and better BNP reduction compared to bolus furosemide alone [11]. Intermittent bI therapy prevents diuretic resistance by preventing excessive sodium reabsorption from the distal tubules. cIV, however, offers a more stable diuretic exposure to the tubule, potentially decreasing the rebound phenomenon and ensuring consistent diuresis. HSS augments furosemide's effect by temporarily increasing serum sodium concentration, leading to sustained sodium delivery to the tubular lumen, potentially reducing diuretic resistance. However, administering HSS without furosemide might have adverse effects due to increased NaCl concentrations, potentially triggering vasoconstriction. Studies evaluating various HSS concentrations have reported differing effects on heart failure patients [12]. Our study opted for a standardized HSS concentration of 1.95% NaCl as all our patients had normal serum sodium levels, ensuring consistent treatment.

In contrast to prior studies, the current trial did not administer bolus furosemide before initiating continuous intravenous furosemide therapy. This difference might have masked the potential superiority of cIV therapy due to the lower achieved furosemide concentrations. Interestingly, our trial displayed a trend, albeit nonsignificant, toward renal function improvement in the HSS group. The administration of infusion of frusemide has shown to be an effective method for mitigating the adverse effects of neurohormonal stimulation and renal function decline. Frusemide infusion rapidly increases extracellular NaCl concentration, enhancing osmotic pressure, facilitating swift fluid mobilization into the vascular compartment, and enhancing renal blood flow. Studies have highlighted renal failure not only as a predictor of heart failure severity but also as a contributing factor in the progression of heart failure [13, 14, 15, 16]. The association between worsening renal function and adverse outcomes has been consistently observed. Thus, an effective diuretic regimen should promptly alleviate congestion without compromising renal function. The cIV of frusemide has been deemed safer for renal function in heart failure patients, particularly, those with extensive edema and diuretic resistance, even in the absence of hyponatremia or hypoalbuminemia. In our study, the group receiving HSS exhibited a slight increase in creatinine levels. This could potentially be attributed to the shorter duration (48 h) and lower concentration of HSS (1.95% NaCl) used compared to other studies, which might have limited the favorable effects of HSS on renal function. However, the HSS regimen might prove more beneficial for diuretic treatment beyond 48 h when its favorable effects on renal function become apparent. Paterna et al. demonstrated a significant reduction in hospital stay with HSS treatment in their study, albeit without a significant difference in weight loss between the furosemide plus HSS regimen and furosemide alone [11]. Similarly, in our study, although the hospital stay was shorter in the cIV group, the weight loss was only insignificantly higher, possibly due to the relatively small sample size.

## 5 Limitations

The study has several limitations that need to be considered when interpreting its findings. First, the relatively small sample size and the single‐center nature of the study might restrict the generalizability of the results to broader populations or different healthcare settings. This limitation suggests the necessity for larger multicenter studies to validate these findings across diverse patient groups. Another limitation arises from the short duration of observation, particularly, the 48‐h treatment period. This timeframe might not capture the longer‐term effects or variations in patient responses to the different diuretic regimens studied. Additionally, the study included heterogeneous patient characteristics, encompassing individuals with both reduced and preserved LVEF, which might have influenced treatment responses and outcomes. Variability in furosemide dosing and administration methods represents another limitation. While the study investigated different modes of furosemide administration, variations in dosages and administration protocols might have impacted the comparative outcomes, potentially influencing the observed results. The exclusion criteria applied in the study, such as pre‐hospital intravenous diuretic use, specific serum creatinine levels, and systolic blood pressure limitations, might introduce selection bias, potentially excluding certain patient groups and limiting the study's applicability to a broader heart failure population. Moreover, the assessment of renal function primarily relied on serum creatinine levels. The absence of a more comprehensive evaluation of renal function parameters could limit the study's ability to provide a thorough understanding of renal outcomes associated with the different diuretic regimens. Additionally, the lack of blinding in the study, where treating physicians were not blinded to the diuretic regimens, might introduce bias in treatment decisions and assessments of patient outcomes. Finally, potential publication bias should be acknowledged, as studies with nonsignificant findings or smaller effect sizes may be less likely to be published, potentially influencing the overall literature on this topic.

## 6 Conclusion

In conclusion, our study comparing diuretic regimens in ADHF highlights notable advantages of continuous intravenous furosemide infusion. While no significant differences were observed in renal function or fluid removal among continuous intravenous furosemide infusion, bolus furosemide injection, and furosemide plus HSS groups, the intravenous bolus without HSS exhibited a notably shorter hospital stay. Further large‐scale studies with longer follow‐up periods are essential to validate these outcomes and delineate optimal diuretic strategies for managing ADHF.

## Conflicts of Interest

The authors declare no conflicts of interest.

## Data Availability

The data that support the findings of this study are available on request from the corresponding author. The data are not publicly available due to privacy or ethical restrictions.
